# Considerations for the future development of virtual technology as a rehabilitation tool

**DOI:** 10.1186/1743-0003-1-13

**Published:** 2004-12-23

**Authors:** Robert V Kenyon, Jason Leigh, Emily A Keshner

**Affiliations:** 1 Electronic Visualization Lab, Department of Computer Science, University of Illinois at Chicago, Chicago, IL, USA; 2 Sensory Motor Performance Program, Rehabilitation Institute of Chicago, Chicago, IL, USA; 3 Department of Physical Medicine and Rehabilitation, Feinberg School of Medicine, Northwestern University, Chicago, IL, USA

**Keywords:** Networking, Rehabilitation, Virtual Reality, Field of View, Complex Behaviors

## Abstract

**Background:**

Virtual environments (VE) are a powerful tool for various forms of rehabilitation. Coupling VE with high-speed networking [Tele-Immersion] that approaches speeds of 100 Gb/sec can greatly expand its influence in rehabilitation. Accordingly, these new networks will permit various peripherals attached to computers on this network to be connected and to act as fast as if connected to a local PC. This innovation may soon allow the development of previously unheard of networked rehabilitation systems. Rapid advances in this technology need to be coupled with an understanding of how human behavior is affected when immersed in the VE.

**Methods:**

This paper will discuss various forms of VE that are currently available for rehabilitation. The characteristic of these new networks and examine how such networks might be used for extending the rehabilitation clinic to remote areas will be explained. In addition, we will present data from an immersive dynamic virtual environment united with motion of a posture platform to record biomechanical and physiological responses to combined visual, vestibular, and proprioceptive inputs. A 6 degree-of-freedom force plate provides measurements of moments exerted on the base of support. Kinematic data from the head, trunk, and lower limb was collected using 3-D video motion analysis.

**Results:**

Our data suggest that when there is a confluence of meaningful inputs, neither vision, vestibular, or proprioceptive inputs are suppressed in healthy adults; the postural response is modulated by all existing sensory signals in a non-additive fashion. Individual perception of the sensory structure appears to be a significant component of the response to these protocols and underlies much of the observed response variability.

**Conclusion:**

The ability to provide new technology for rehabilitation services is emerging as an important option for clinicians and patients. The use of data mining software would help analyze the incoming data to provide both the patient and the therapist with evaluation of the current treatment and modifications needed for future therapies. Quantification of individual perceptual styles in the VE will support development of individualized treatment programs. The virtual environment can be a valuable tool for therapeutic interventions that require adaptation to complex, multimodal environments.

## Background

Visual imaging is one of the major technological advances of the last decade. Although its impact in medicine and research is most strongly observed in the explosion of PET and fMRI studies in recent years [1], there has been a steady emergence of studies using virtual imaging to measure and train human behavior. Virtual environments (VE) or virtual reality (VR) have taken a foot hold in rehabilitation with dramatic results in some cases. Some applications have the patient wearing VE systems to improve their ability to locomote [2]. Others bring the VE technology to the patient to improve much needed rehabilitation [3]. With either approach, there are at least two issues that need to be addressed by the clinical or basic scientist employing virtual technology to elicit natural human behaviors. One is the ability of the technology to present images in real-time. If the virtual stimulus has delays that exceed those expected by the central nervous system (CNS), then the stimulus will most likely be ignored or processed differently than inputs from the physical world. Once a response is elicited, it must be determined whether the variability observed across individuals is due to individual differences or inconsistencies between expectation and the presentation of the virtual image.

## Components of a virtual environment

Let us first define what we consider a VE and consider the signals that need to be transmitted for such a system to operate remotely (TeleImmersion). VE is immersion of a person in a computer generated environment such that the person experiences stereovision, correct perspective for all objects regardless of their motion, and objects in the environment move in a natural fashion with subject motion. To achieve theses characteristics, certain technology must be utilized. To provide stereovision, slightly different images must be presented to the right and left eyes with little if any cross talk between the two images. In some systems this is provided by using field sequential stereo in combination with liquid crystal shutter glasses (StereoGraphics, Inc). In this system the right liquid crystal lens is clear while the left is opaque and the perspective scene generated on the screen is that for the right eye. Then the left eye lens is clear and the right is opaque and the left eye's view is displayed. This method of producing stereo has found its way into projection based systems [4,5] and desktop systems also known as "fish tank VR" [6]. In other systems the person wears a head mounted display (HMD) where the right and left eye each see a dedicated display so that the computer generates a left and right eye perspective image and each image is connected to the corresponding monitor. Such systems have used miniature CRTs, Liquid Crystal Displays, and Laser light directed into the eye to create the image on the retina [7]. In contrast to the above mentioned systems, an auto-stereographic system displays stereo images to the person without the aid of any visual apparatus worn by the person [8]. The person merely looks at the screen(s) and sees stereo images as one might in the natural world. Because of their ease of use by the subject and their versatility these new and experimental systems have the potential of becoming the ultimate VE display when large motions of the subject are not needed.

Regardless of the system used, to keep all the stereo objects in the correct perspective and to keep them from being distorted when the person moves in the environment, it is necessary to track the movements of the person so that the computer can calculate a new perspective image given the reported location of the person's head/eyes. The tracking systems that are used to do this are varied. The most commonly used of these are the 6-degrees of freedom (DOF) magnetic tracking systems (Ascension, Inc and Polhemus, Inc.). With these systems a small sensor cube is placed on the subject and the location of the sensor within the magnetic field is detected. When the sensor is place on the head or glasses of the person the orientation of the head and therefore the location of the eyes can be presumed. Other non-magnetically based systems use a combination of acoustic location to delineate position and acceleration detection to obtain body coordinates in space. The combination results in 6 DOF for the location information (InterSense, Inc). Other systems use cameras to track the person and then transform this information to the 6-DOF needed to maintain a proper image in the VE (Motion Analysis, Inc).

So far we have confined our discussion to visual objects and have not considered the use of haptic or other forms of information to be integrated into the VE system [9]. To provide a realistic haptic experience to the subject, objects must be rendered at 1000 times per second. While a local haptic system such as that produced by Sensable Inc. and others can provide such high speed communication, when such information is floated over the network the issues of bandwidth and latency of the network are paramount to consider. While experimental networks have significantly increased the bandwidth of the network, our ability to move information over these networks is currently fixed by the speed of light. Prediction and other methods can be employed to help reduce the effective latency (Handshake Technologies, Inc), but this characteristic will continue to pose a problem for many conditions that we would like to use in tele-rehabilitation.

In networked VEs several types of data need to be transmitted between collaborating sites: 1. the main data-set itself (this often consists of 3D geometry); 2. the changes to the data-set (these occur when collaborating users modify the geometry in some way – perhaps by moving the object or deforming it); 3. the virtual representation of the remote collaborator (this often is referred to as an avatar); 4. the video and/or audio channel (that facilitates face-to-face conversation.) Video has limited use in stereoscopic projection-based VEs because the large shutter glasses that the viewer uses to resolve the stereo tends to hide the viewers face from the camera. Furthermore most stereoscopic projection systems operate in dimly lit rooms which are usually too dark for effective use of video.

The common model for data sharing in networked VEs is to have most of the main data-set replicated across all the sites and transmit only incremental changes. Furthermore the main data-set is often cached locally at each of the collaborating sites to reduce the need for having to retransmit the entire data-set each time the application is started. Classically TCP (Transmission Control Protocol – the protocol that is widely used on the Internet for reliable data delivery) has been the default protocol used to distribute the data-sets. TCP works well in low-bandwidth (below 10 Mb/s) or short distance (local area) networks. However for high-bandwidth long-distance networks, TCP's conservative transmission policy thwarts an application's attempt to move data expediently, regardless of the amount of bandwidth available on the network. This problem is known as the Long Fat Network (LFN) problem [10]. There are a wide variety of solutions to this [11], however none of them have been universally adopted.

Changes made to the 3D environment need to be propagated with absolute reliability and with minimal latency and jitter. Latency is the time it takes for a transmitted message to reach its destination. Jitter is the variation in the latency. Fully reliable protocols like TCP have too much latency and jitter because the protocol requires an acknowledgment to verify delivery. Park and Kenyon [12] have shown that jitter is far more offensive than latency. One can trade off some latency for jitter by creating a receiving buffer to smooth out the incoming data stream. UDP (User Datagram Protocol) on the other hand transmits data with low latency and jitter, but is unreliable. Forward Error Correct (FEC) is a protocol that uses UDP to attempt to correct for transmission errors without requiring the receiver to acknowledge the sender. FEC works by transmitting a number of redundant data packets so that if one is lost at the receiving end, the missing data can be reconstructed from the redundant packets [13]. FEC however is not completely reliable. Hence to achieve complete reliability (at the expense of an infrequent increase in jitter) FEC is often augmented with an acknowledgment mechanism that is only used when it is unable to reconstruct a missing packet.

The virtual representation of a remote collaborator (avatar) is often captured as the position and orientation of the 3D tracking devices that are attached to the stereoscopic glasses and/or 3D input device (e.g. a wand). With simple inverse kinematics one is able to map this position and orientation information onto a 3D geometric puppet, creating lifelike movements [14]. The 3D tracking information is often transmitted using UDP to minimize latency and jitter – however since the data is mainly used to convey a user's gesture, absolute delivery of the data is not necessary. Furthermore since tracking data is transmitted as an un-ending stream, a lost packet is often followed soon after (usually within 1/30^th ^of a second) by a more recent update.

Audio and video data are similar in property to the avatar data in that they usually comprise an unending stream that is best transmitted via UDP to minimize latency and jitter. Often video and audio packets are time stamped so that they can be synchronized on the receiving end. When more than two sites are involved in collaboration it is more economical to send audio/video via multicast. In multicast the sender sends the data to a specific device or machine that then copies the data to the various people that are subscribers to the data. For example, a user send their data to a multicast address and the routers that receive the data send copies of the data to remote sites that are subscribed to the multicast address. One drawback of multicast is that it is often disabled on routers on the Internet as one can potentially inundate the entire Internet. An alternative approach is to use dedicated computers as "repeaters" that intercept packets and transmit copies only to receivers that are specifically registered with the repeater. This broadcast method tends to increase the latency and jitter of packets, especially as the number of collaborators increases.

### Quality of Service (QoS)

QoS refers to a network's ability to provide bandwidth and/or latency guarantees. QoS is crucial for applications such as networked VE, especially those involving haptics or tele-surgery, which are highly intolerant of latency and jitter. Early attempts to provide QoS (such as Integrated Services and Differentiated Services) have been good research prototypes but have completely failed to deploy across the wider Internet because telecommunications companies are not motivated to abide by each others QoS policies. It has been argued that QoS is unnecessary because in the future all the networks will be over-provisioned so that congestion or data loss that result in latency and jitter, will never occur. This has been found to be untrue in practice. Even with the enormous increase in bandwidth accrued during the dot-com explosion, the networks are still as unpredictable as they were a decade ago. Ample evidence is available from the online gaming community which often remarks about problems with bandwidth, latency and jitter during game sessions [15]. These games are based on the same principles that govern the design of networked VEs and therefore serve as a good metric for the current Internet's ability to support tightly coupled collaborative work.

### Customer Owned Networks

Frustrated by the lack of QoS on the Internet, there is growing interest in bypassing the traditional routed Internet by using the available dark fiber in the ground. Dark fiber is optical fiber that has not yet been lit. Currently it is estimated that only about 5–10% of the available fiber has been lit, and each fiber has several terabits/s of capacity. The dot-com implosion has made this dark fiber and wavelengths of light in the fiber, very affordable. The newly emerging model is to construct a separate customer-owned network by purchasing or leasing the fiber from a telecommunications company, and installing one's own networking equipment at the endpoints. A number of federally supported national and international initiatives have been underway for the last few years to create customer-controlled networks explicitly for the scientific community. These include the National Lambda rail [16], StarLight [17], and the Global Lambda Integrated Facility [18]. By creating dedicated fiber networks, applications will be able to schedule dedicated and secure light paths with tens of gigabits/s of unshared, uncongested bandwidth between collaborating sites. This is the best operating environment for tightly coupled networked, haptic VEs.

### Connection Characteristics for Rehabilitation

The ability to use virtual technology for rehabilitation is a function of cost, availability, and the kind of applications that can best utilize the network and provide rehabilitation services. Thus far, tele-rehabilitation research has focused on the use of low speed and inexpensive communication networks. While this work is important, the potential of new high-speed networks has not gathered as much attention. Consequently, we have little but imagined scenarios of how such networks might be utilized. Let us consider the case where a high-speed network connects a rehabilitation center and a remote clinic. The question is what kind of services can be provided remotely.

**Figure 1:**
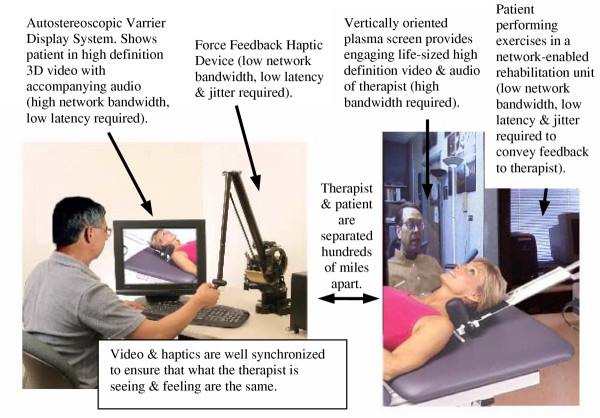
Possible tele-rehabilitation scenario facilitated by high bandwidth networking.

The scenario that we envision is one where patients are required to appear at a rehabilitation center to receive therapy. Our scenario could work in several conditions. For example, a therapist at one location may want an opinion about the patient from a colleague at another location or, perhaps, the therapist can only visit the remote location once per week and with virtual technology the daily therapy could still be monitored by the therapist remotely. In our imagined condition we have a therapist at a rehabilitation center with VE, haptic and video devices and software to help analyze the incoming data (i.e., data mining) feeding to a remote clinic with identical equipment connected together through a dedicated high speed network. As displayed in Fig. 1, the therapist station has several areas of information that connects him/her to the patient in the remote clinic. The VE (in this case Varrier) provides the therapist with a representation of the patient and the kind of trajectory that will be needed for this training session. Notice that the use of Varrier removes the need for HMD or shutter glasses to be worn by the patient or therapist. This may seem like a minor difference, but now the patient and the therapist can see each other eye to eye. The video connection allows more communication (non-verbal or bed side manner) to take place between the two linked users of this system. The haptic device serves two purposes (1) to feedback the forces from the patient's limb to the therapist and (2) to feed the forces that the therapist wishes the patient to experience. Furthermore, we could provide a task that uses the affected limb so that learning and coordination is encouraged. Other possibilities include having the robot apply forces to the patient appendage so that adaptation and recovery of function occurs [9]. In our scenario we could allow the patient to see both the virtual limb and their own limb if needed by the therapy. As can be seen from Fig. 1, the bandwidth and latency requirements change as a function of the kind of information that is being transmitted.

A system as described above is possible today although expensive. The network characteristics that would be needed for each information channel would be as follows. A high-bandwidth connection would be needed for video and audio streamed to the plasma displays at each location, in addition to the high bandwidth a low latency and jitter connection would be needed for the Varrier Display system (VE). For a force feedback haptic device communicating between the patient and the therapist, a low network bandwidth could be used but the latency and jitter need to be low.

## Response behaviors in the virtual environment

After all possible consideration of how to best construct the virtual system, the next concern is how to associate the complex stimuli with the behavior of interest. The relative influence of particular scene characteristics, namely field of view (FOV), scene resolution, and scene content, are critical to our understanding of the effects of the VE on our response behaviors [19] and the effect of these characteristics on postural stability in an immersive environment has been examined [20]. Roll oscillations of the visual scene were presented at a low frequency – 0.05 Hz to 10 healthy adult subjects. The peak angular velocity of the scene was approximately 70°/sec. Three different scenes (600 dpi fountain scene, 600 dpi simple scene, and 256 dpi fountain scene) were presented at 6 different FOVs (+/-15°, 30°, 45°, 60°, 75°, 90° from the center of the visual field) counterbalanced across subjects. Subjects stood on a force platform, one foot in front of the other, with their arms crossed behind their backs. Data collected for each trial included stance break (yes, no), latency to stance break (10 sec maximum), subjective difficulty rating (difficulty in maintaining the Romberg stance, 1–10 scale), and dispersion of center-of-balance. Postural stability was found to vary as a function of display FOV, resolution, and scene content. Subjects exhibited more balance disturbance with increasing FOVs, higher resolutions and more complex scene contents. Thus, altered scene contents, levels of interactivity, and resolution in immersive environments will interact with the FOV in creating a postural disturbance.

Expectation of the visual scene characteristics will also influence responses in a VE. When subjects had some knowledge of the characteristics of a forthcoming visual displacement most reduced their postural readjustments, even when they did not exert active control over the visual motion [21]. Thus we can hypothesize that visual stimuli present an optimal pathway for central control of postural orientation as there are many cues in the visual flow field that can identified for anticipatory processing. The important parameters of the visual field on posture can be extracted from several studies. Vestibular deficient individuals who were able to stabilize sway when fixating on a stationary light [22] became unstable when an optokinetic stimulus was introduced, implying that velocity information from peripheral vision was a cause of instability. Focusing upon distant visual objects in the environment increased postural stability [23,24]. We have observed in the VE [25,26] that small physical motions combined with large visual stimuli trigger a perception of large physical movements as occurs during flight simulations [27] and gaming. We have also observed measurable increases in the variability of head and trunk coordination and increased lateral head and trunk motion when standing quietly and walking within a dynamic visual environment [28].

The challenge is to determine whether the subject has become immersed in the environment, i.e., has established a sense of presence in the environment (see paper by Riva in this issue), and then to establish the correlation between the stimulus and response properties. The experience within the VE is multimodal, requiring participation of all sensory pathways as well as anticipatory processing and higher order decision making. Consequently, it is difficult to attribute resultant behaviors to any single event in the environment and responses across participants may be very variable. We have united an immersive dynamic virtual environment with motion of a posture platform [25] to record biomechanical and physiological responses to combined visual, vestibular, and proprioceptive inputs in order to determine the relative weighting of physical and visual stimuli on the postural responses.

## Methods

In our laboratory, a linear accelerator (sled) that could be translated in the anterior-posterior direction was controlled by D/A outputs from an on-line PC. The sled was placed 40 cm in front of a screen on which a virtual image was projected via a stereo-capable projector (Electrohome Marquis 8500) mounted behind the back-projection screen. The wall in our system consisted of back projection material measuring 1.2 m × 1.6 m. An Electrohome Marquis 8500 projector throws a full-color stereo workstation field (1024 × 768 stereo) at 200 Hz [maximum] onto the screen. A dual Pentum IV PC with a nVidia 900 graphics card created the imagery projected onto the wall. The field sequential stereo images generated by the PC were separated into right and left eye images using liquid crystal stereo shutter glasses worn by the subject (Crystal Eyes, StereoGraphics Inc.). The shutter glasses limited the subject's horizontal FOV to 100° of binocular vision and 55° for the vertical direction. The correct perspective and stereo projections for the scene were computed using values for the current orientation of the head supplied by a position sensor (Flock of Birds, Ascension Inc.) attached to the stereo shutter glasses (head). Consequently, virtual objects retained their true perspective and position in space regardless of the subjects' movement. The total display system latency from the time a subject moved to the time the new stereo image was displayed in the environment was 20–35 ms. The stereo update rate of the scene (how quickly a new image is generated by the graphics computer in the frame buffer) was 60 stereo frames/sec. Flock of birds data was sampled at 120 Hz.

### Scene Characteristics

**Figure 2:**
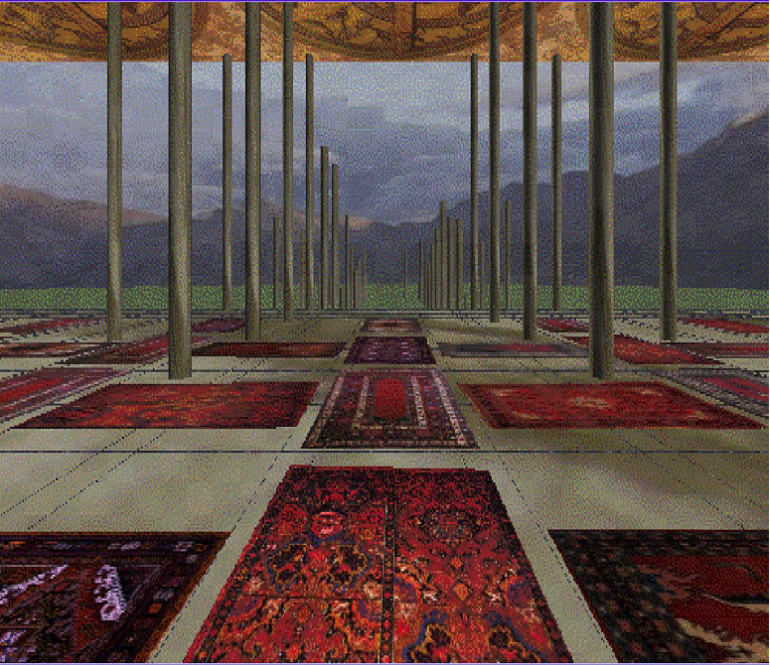
An illustration of the virtual environment image in our laboratory.

The scene consisted of a room containing round columns with patterned rugs and painted ceiling (Fig. 2). The columns were 6.1 m apart and rose 6.1 m off the floor to the ceiling. The rug patterns were texture mapped on the floor and consisted of 10 different patterns. The interior of the room measured 30.5 m wide by 6.1 m high by 30.5 m deep. The subject was placed in the center of the room between two rows of columns. Since the sled was 64.8 cm above the laboratory floor the image of the virtual room was adjusted so that its height matched the sled height (i.e., the virtual floor and the top of the sled were coincident). Beyond the virtual room was a landscape consisting of mountains, meadows, sky and clouds. The floor was the distance from the subject's eyes to the virtual floor and the nearest column was 4.6 m away. The resolution of the image was 7.4 min of arc per pixel when the subject was 40 cm from the screen. The view from the subjects' position was that objects in the room were both in front of and behind the screen. When the scene moved in fore-aft, objects moved in and out of view depending on their position in the scene.

### Procedures

Subjects gave informed consent according to the guidelines of the Institutional Review Board of Northwestern University Medical School to participate in this study. Subjects had no history of central or peripheral neurological disorders or problems related to movements of the spinal column (e.g., significant arthritis or musculoskeletal abnormalities) and a minimum of 20/40 corrected vision. All subjects were naive to the VE.

We have tested 7 healthy young adults (aged 25–38 yrs) standing on the force platform (sled) with their hands crossed over their chest and their feet together in front of a screen on which a virtual image was projected. Either the support surface translated ± 15.7 cm/sec (± 10 cm displacement) in the a-p direction at 0.25 Hz, or the scene moved ± 3.8 m/sec (± 6.1 m displacement) fore-aft at 0.1 Hz, or both were translated at the same time for 205 sec. Trials were randomized for order. In all trials, 20 sec of data was collected before scene or sled motion began (pre-perturbation period). When only the sled was translated, the visual scene was visible but stationary, thus providing appropriate visual feedback equivalent to a stationary environment.

### Data Collection and Analysis

Three-dimensional kinematic data from the head, trunk, and lower limb were collected at 120 Hz using video motion analysis (Optotrak, Northern Digital Inc., Ontario, Canada). Infrared markers placed near the lower border of the left eye socket and the external auditory meatus of the ear (corresponding to the relative axis of rotation between the head and the upper part of the cervical spine) were used to define the Frankfort plane and to calculate head position. Other markers were placed on the back of the neck at the level of C7, the left greater trochanter, the left lateral femoral condyle, the left lateral malleolus, and on the translated surface. Markers placed at C7 and the greater trocanter were used to calculate trunk position, and shank position was the calculated from the markers on the lateral femoral condyle and the lateral malleolus.

For trials where the sled moved, sled motion was subtracted from the linear motion of each segment prior to calculating segmental motion. Motion of the three segments was presented as relative segmental angles where motion of the trunk was removed from motion of the head to determine the motion of the head with respect to the trunk. Motion of the shank was removed from motion of the trunk to reveal motion of the trunk with respect to the shank. Motion of the shank was calculated with respect to the sled.

## Results

**Figure 3:**
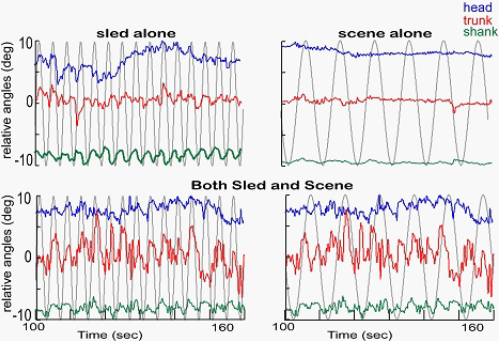
Relative angles of the head to trunk (blue), trunk to shank (red) and shank to sled (green) are plotted for a 60 sec period of the trial during sled motion only, scene motion only, and combined sled and scene motion (the same data are plotted against both the sled and the scene).

The response to visual information was strongly potentiated by the presence of physical motion. Either stimulus alone produced marginal responses in most subjects. When combined, the response to visual stimulation was dramatically enhanced (Fig. 3), perhaps because the visual inputs were incongruent with those of the physical motion.

**Figure 4:**
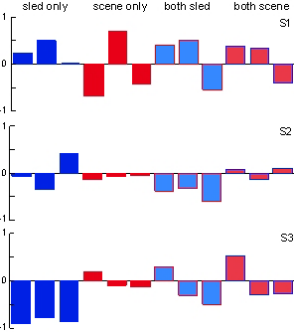
Overall weighting of the input variables derived from the PCA for 3 subjects. The first 3 bars (blue) represent a subsequent non-overlapping 40 sec time period to sled motion only. The next 3 bars (red) represent non-overlapping 40 sec time periods to scene motion only. The last 6 bars represent non-overlapping 40 sec time periods to both sled (blue) and scene (red) motion. The direction of each bar indicates the relative phase between the response and the input signal.

Using Principal Component Analysis we have determined the overall weighting of the input variables. In healthy young adults, some subjects consistently responded more robustly when receiving a single input, suggesting a proprioceptive (see S3 in Fig. 4) or visual (S1 in Fig. 4) dominance. With multiple inputs, most subjects produced fluctuating behaviors so that their response was divided between both inputs. The relative weighting of each input fluctuated across a trial. When the contribution of each body segment to the overall response strategy was calculated, movement was observed primarily in the trunk and shank.

## Discussion

Results from experiments in our laboratory using this sophisticated technology revealed a non-additive effect in the energy of the response with combined inputs. With single inputs, some subjects consistently selected a single segmental strategy. With multiple inputs, most produced fluctuating behaviors. Thus, individual perception of the sensory structure was a significant component of the postural response in the VE. By quantifying the relative sensory weighting of each individual's behavior in the VE, we should be better able to design individualized treatment plans to match their particular motor learning style. Developing treatment interventions in the virtual environment should carry over into the physical world so that functional independence will be increased for many individuals with physical limitations. In fact, there is evidence that the knowledge and skills acquired by disabled individuals in simulated environments can transfer to the real world [29-31].

The ability for us to use this technology outside the area of research labs and bring these systems to clinics is just starting. However, the cost is high and the applications that can best be applied to rehabilitation are limited. The cost of such systems might be mitigated if this technology allowed therapists and patients to interact more frequently and/or resulted in better patient outcomes. Such issues are under study now at several institutions. This brings us to the idea of tele-rehabilitation, which would allow therapy to transcend the physical boundaries of the clinic and go wherever the communication system and the technology would allow [5]. For example, at some location remote from the clinic a patient enters a VE suitable for rehabilitation protocols connected to the clinic and a therapist. While this idea is not new, the kind of therapies that could be applied under such a condition is limited by the communication connection and facilities at both ends of the communication cable.

The ability to provide rehabilitation services to locations outside the clinic will be an important option for clinicians and patients in the near future. Effective therapy may best be supplied by the use of high technology systems such as VE and video, coupled to robots, and linked between locations by high-speed, low-latency, high-bandwidth networks. The use of data mining software would help analyze the incoming data to provide both the patient and the therapist with evaluation of the current treatment and modifications needed for future therapies.

## Conclusions

The ability to provide rehabilitation services to locations outside the clinic is emerging as an important option for clinicians and patients. Effective therapy may best be supplied by the use of high technology systems such as VE and video, coupled to robots, and linked between locations by high-speed, low-latency, high-bandwidth networks. The use of data mining software would help analyze the incoming data to provide both the patient and the therapist with evaluation of the current treatment and modifications needed for future therapies. Although responses in the VE can vary significantly between individuals, these results can actually be used to benefit patients through the development of individualized treatments programs that will raise the level of successful rehabilitative outcomes. Further funding for research in this area will be needed to answer the questions that arise from the use of these technologies.
